# Long Term Follow-up of an Open Bicondylar Hoffa Fracture with a Disrupted Extensor Mechanism: A Case Report

**DOI:** 10.5704/MOJ.1907.013

**Published:** 2019-07

**Authors:** CM Joseph, YS Rama-Prasad, PRJVC Boopalan, TS Jepegnanam

**Affiliations:** Department of Orthopaedics, Christian Medical College, Vellore, India; * Department of Orthopaedics, Amulya hospital, Visakhapatnam, India; ** >Department of Orthopaedics, Christian Medical College, Vellore, India

**Keywords:** long term, bicondylar hoffa, open fracture, comminuted patella, outcome

## Abstract

This is the first report of a long-term follow-up of an open bicondylar Hoffa with patella fracture. It is interesting to note the radiological changes of osteoarthritis 15 years after global intra-articular injury of the distal femur. The good clinical outcome is possibly due to the integrity of the knee ligaments and reconstruction of the extensor mechanism in addition to stable anatomical reduction and fixation.

## Introduction

Anatomical reduction and surgical fixation of Hoffa fractures seem to show good results in the short term^1,2^. However long-term outcomes may be poorer. Few studies have been published on bicondylar Hoffa fractures demonstrating satisfactory to good outcomes at follow-ups ranging from one to three years^1,3,4^. There have been no longterm reports of this injury. We describe a young patient who sustained an open bicondylar Hoffa and comminuted patella fracture at a 15-year follow-up.

## Case Report

**Fig. 1:**
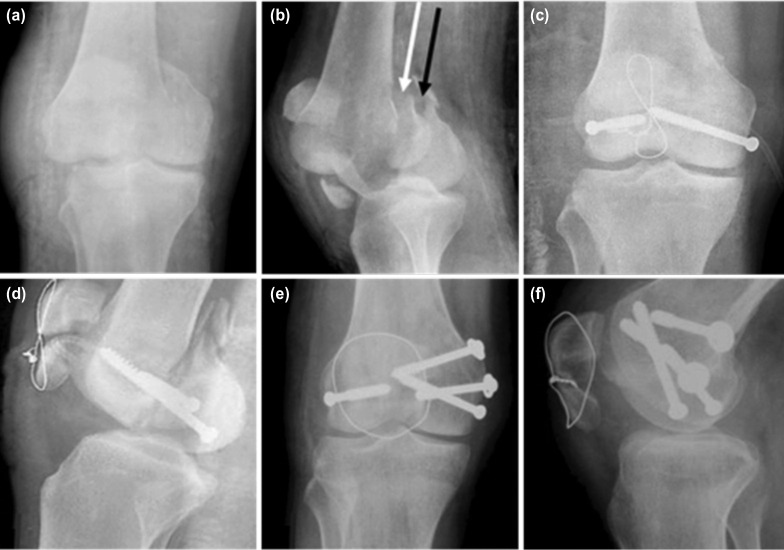
Radiographs at initial presentation, (a) Antero-posterior (AP) and (b) lateral view of the right knee showing the bicondylar Hoffa fracture (black and white arrow point to the larger medial condyle and small lateral condyle fragment respectively). Radiographs following anatomical reduction and stable internal fixation with 3 partially threaded 6.5mm cancellous lag screws with 2 washers for the medial condyle and 1 lag screw for the lateral condyle combined with a patella cerclage wire. Immediate postoperative (c) AP and (d) Lateral view of the right knee. (e) AP and (f) lateral view of the right knee at three months following surgery showing well united bicondylar Hoffa and patella fractures.

A 20-year old gentleman was injured in a high velocity road traffic accident while riding a motorcycle. A sheet of metal went through his right knee. He was brought to the emergency department nine hours following the trauma. He had an 8 x 6cm grossly contaminated transverse laceration over the anterior aspect of his knee exposing a comminuted fractured patella and radiographs further revealed a bicondylar Hoffa (AO 33-B3) fracture (Fig. 1(a) and (b)). He had no distal neurovascular deficits.

He underwent emergency debridement and removal of the loose patella fragments. The laceration was extended and the distal femur was visualised through the patella fracture. The bicondylar fracture was found to have a large medial and a smaller lateral condyle fragments. The quadriceps, patella tendon, collateral, cruciate ligaments and menisci were found to be intact. Minimal internal fixation was done in the initial stage in view of the contaminated nature of the injury. The bicondylar Hoffa fracture was stabilised with two posteroanterior lag screws (Synthes 6.5mm partially threaded cancellous screws), one for each condyle along with a cerclage 16-gauge wire for the patella (Fig. 1(c) and (d)). At elective relook debridement 48 hours later, two more lag screws (Synthes 6.5mm partially threaded cancellous screws with washers) were added for the larger medial condyle fragment. The fractures were found to be stable following fixation.

**Fig. 2:**
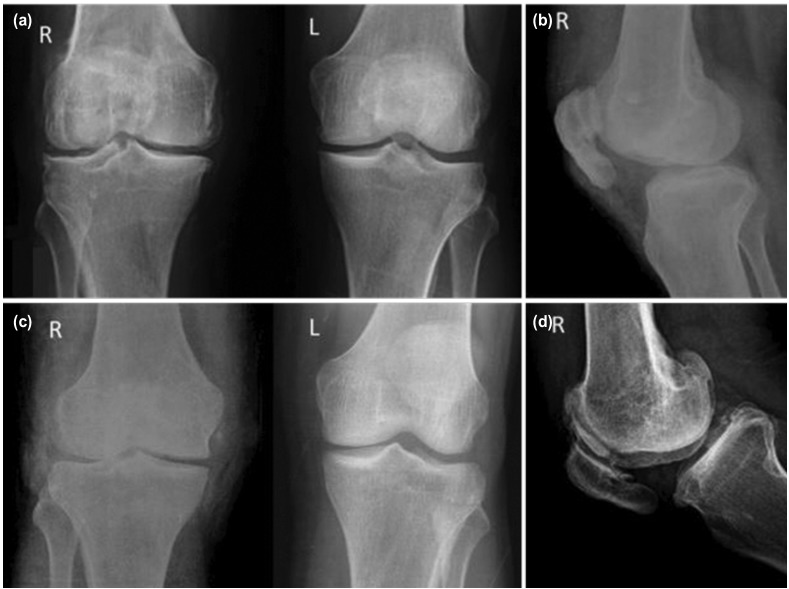
At one month following implant exit, (a) AP view of both knees (R-Right, L-Left) and (b) lateral view of the right knee with no visible arthritic changes in the right knee. At final follow-up of 15 years, (c) AP view of both knees and (d) lateral view of the right knee showing osteoarthritic changes in the right knee (osteophytes around the patella and both femoral condyles).

The wound healed uneventfully and he was immobilised in an above knee cast for six weeks in view of the comminuted patella fracture. He was subsequently started on active knee range of movement (ROM) exercises and graduated weight bearing. At three months, he underwent manipulation under anesthesia as he had some residual knee stiffness with flexion up to 90^°.^ Following manipulation, his ROM improved and he was advised regular follow-up. Three years later, he presented with anterior knee pain and implant prominence over the knee. It was then decided on implant exit for the patella. However, in view of the extensive intra-articular extent of the fracture, unavoidable consequences like knee stiffness and early arthritis in the future, the distal femur hardware was removed in the same sitting. Radiographs following implant exit showed a well-united fracture without arthritic changes in the knee (Fig. 2(a) and (b)).

**Fig. 3:**
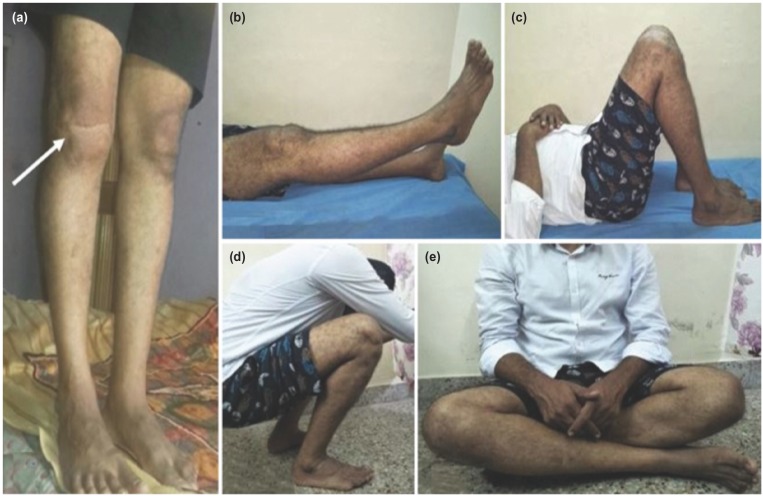
At final clinical follow-up of 15 years showing the patient, (a) standing with both knees in alignment (white arrow pointing to the right knee with a healed transverse laceration), (b) actively extending his right knee till 0^°^ with no lag, (c) with right knee flexion of 130^°,^ (d) squatting and (e) sitting cross-legged.

As this was an open intra-articular fracture, he was advised clinical follow-up of atleast once in three years following union of the fracture. He was very compliant and was on regular visits. At latest follow-up (15 years since the trauma), he was asymptomatic, although there were Kelgren-Lawrence grade 2 osteoarthritic changes of the right knee visible on radiographs (Fig. 2(c) and (d)). The gait was normal and both knees were in alignment (Fig. 3(a)). He had no pain or extensor lag and his knee was clinically stable with a flexion of 130^°^ which was 5^°^ short of the normal side (Fig. 3(b) and (c)). He could squat, sit cross-legged (Fig. 3(d) and (e)) and had resumed his normal level of activity which included playing badminton at the club level. His Oxford Knee Score was 47 and his knee and functional score components of the Knee Society Score were 85 and 100 respectively.

## Discussion

This case report describes an excellent long-term functional outcome for an open bicondylar Hoffa fracture with a disrupted extensor mechanism. These fractures are extremely rare and complications such as non-union, implant failure, arthrofibrosis and osteoarthritis are not uncommon^1^. Anatomical reduction, stable fixation and early functional rehabilitation are the mainstay of management.

Of the few described cases in literature, most required intra-articular arthrolysis to regain knee ROM following fixation. Calmet *et al* reported on an open Bicondylar Hoffa fracture case with disrupted extensor mechanism that needed arthroscopic arthrolysis at three months despite immediate knee mobilization postoperatively^4^. Mounasamy *et al* described a case which also had an open transverse patella fracture requiring tension band wiring^3^. During arthroscopic arthrolysis at nine months it was noted that there were grade II/III tri-compartmental osteochondral defects along with a 1.5cm osteochondral lesion in the medial femoral condyle. Our patient had an open comminuted patella fracture as well and required immobilisation for six weeks. At three months, closed manipulation under anesthesia was performed to improve knee ROM due to residual stiffness.

Onay *et al* reported an osteoarthritis rate of 54% in a consecutive series of 13 patients with unicondylar Hoffa fractures at a longer mean follow-up period of 93 months^5^. Literature on the long-term outcomes of bicondylar Hoffa fractures is lacking. Though the gradual progression to early expected arthritis following surgical fixation in this injury cannot be prevented, the presence of intact cruciate and collateral ligaments may have a long-term role in preventing extensive cartilage damage. All available literature on bicondylar fractures with good outcomes in the short term have asserted the integrity of these ligaments^3,4^. In this patient, radiographic changes of knee osteoarthritis were visible at 15 years. We believe that the integrity of the knee ligaments could be a likely contributing factor to the good clinical result.

This study demonstrates that open bicondylar Hoffa fractures, despite being notorious for complications, can still result in an excellent long-term functional outcome. Early debridement, anatomical reduction and stable rigid fixation combined with the integrity of the cruciate and collateral ligaments would appear to be the key in getting a good result in this severe intra-articular fracture.
